# Distinguishing Natural Infections of the Bovine Mammary Gland by *Staphylococcus* from *Streptococcus* spp. Using Quantitative Milk Proteomics

**DOI:** 10.3390/ani13111829

**Published:** 2023-05-31

**Authors:** Dina Rešetar Maslov, Funmilola Clara Thomas, Anđelo Beletić, Josipa Kuleš, Ivana Rubić, Miroslav Benić, Goran Bačić, Nino Maćešić, Vida Eraghi, Vladimir Farkaš, Tihana Lenac Roviš, Berislav Lisnić, Damir Žubčić, Dalibor Potočnjak, Vladimir Mrljak

**Affiliations:** 1 Laboratory of Proteomics, Internal Diseases Clinic, Faculty of Veterinary Medicine, University of Zagreb, Heinzelova Street 55, 10000 Zagreb, Croatia; fthomas@vef.hr (F.C.T.); abeletic@vef.hr (A.B.); irubic@vef.unizg.hr (I.R.); veraghi@vef.hr (V.E.); vfarkas@vef.hr (V.F.); vmrljak@vef.unizg.hr (V.M.); 2 Department of Chemistry and Biochemistry, Faculty of Veterinary Medicine, University of Zagreb, Heinzelova Street 55, 10000 Zagreb, Croatia; jkules@vef.hr; 3 Department of Bacteriology and Parasitology, Croatian Veterinary Institute, Savska Cesta, 143, 10000 Zagreb, Croatia; benic@veinst.hr; 4 Reproduction and Obstetrics Clinic, Faculty of Veterinary Medicine, University of Zagreb, Heinzelova Street 55, 10000 Zagreb, Croatia; bacic@vef.hr (G.B.); nmacesic@vef.unizg.hr (N.M.); 5 Center for Proteomics University of Rijeka, Faculty of Medicine, Brace Branchetta 20, 51000 Rijeka, Croatia; tihana.lenac@medri.uniri.hr (T.L.R.); berislav.lisnic@medri.uniri.hr (B.L.); 6 Internal Diseases Clinic, Faculty of Veterinary Medicine, University of Zagreb, Heinzelova Street 55, 10000 Zagreb, Croatia; dazubcic@vef.unizg.hr (D.Ž.); dpotocnjak@vef.unizg.hr (D.P.)

**Keywords:** *Staphylococcus*, *Streptococcus*, bovine mastitis, proteomics, markers, bacterial intramammary infection

## Abstract

**Simple Summary:**

Detection of new diagnostic markers would greatly influence the institution of preventive (control) protocols in bovine mastitis. Using the quantitative proteomics workflows and statistics for milk analysis, we identified that protein kinase C-binding protein NELL2, thrombospondin-1, and complement factor I have diagnostic potential for differentiating staphylococci and streptococci intramammary natural infection and inflammation, and subsequently gained a deeper understanding of the immunopathology of mastitis-related infection and inflammation for various etiological agents.

**Abstract:**

Bovine mastitis is the most frequent disease on dairy farms, which leads to a decrease in the health welfare of the animals and great economic losses. This study was aimed at determining the quantitative variations in the milk proteome caused by natural infection by *Staphylococcus* and *Streptococcus* species in order to gain further understanding of any discrepancies in pathophysiology and host immune responses, independent of the mastitis level. After identification of *Staphylococcus* (N = 51) and *Streptococcus* (N = 67) spp., tandem mass tag (TMT)-labeled quantitative proteomic and liquid chromatography-mass spectrometry (LC-MS/MS) techniques on a modular Ultimate 3000 RSLCnano system coupled to a Q Exactive Plus was applied on aseptically sampled milk from Holstein cows. Proteome Discoverer was used for protein identification and quantitation through the SEQUEST algorithm. Statistical analysis employing R was used to identify differentially abundant proteins between the groups. Protein classes, functions and functional-association networks were determined using the PANTHER and STRING tools and pathway over-representation using the REACTOME. In total, 156 master bovine proteins were identified (two unique peptides, *p* < 0.05 and FDR < 0.001), and 20 proteins showed significantly discrepant abundance between the genera (*p* < 0.05 and FDR < 0.5). The most discriminatory proteins per group were odorant-binding protein (higher in staphylococci) and fibrinogen beta chain protein (higher in streptococci). The receiver operating characteristic (ROC) curve showed that protein kinase C-binding protein NELL2, thrombospondin-1, and complement factor I have diagnostic potential for differentiating staphylococci and streptococci intramammary infection and inflammation. Improved understanding of the host response mechanisms and recognition of potential biomarkers of specific-pathogen mastitis, which may aid prompt diagnosis for control implementation, are potential benefits of this study.

## 1. Introduction

Bovine mastitis (BM) has been the focus of numerous and extensive research over the years [1,2,3]. A notable target of much of the research is centered on identifying novel biological markers and the development of rapid, sensitive, and specific diagnostic assays [2]. Prompt, comprehensive diagnosis, which includes the detection of pathogens causing intramammary infection and inflammation, is crucial for assisting early treatment decisions, especially concerning the choice of antimicrobials’ usage [4]. With the growing threat of antibiotic resistance, guided stewardship in administration and the reduction of these agents have become critical also in dairy establishments. Control and prevention of udder infection become principal factors for the improvement of animal health and well-being on dairy farms, contributing to the reduction of mastitis cases and economic losses [5]. In addition to gaining a deeper understanding of the immunopathology of mastitis-related infection and inflammation for various etiological agents [6], the etiological differentiation of mastitis pathogens would greatly affect the institution of preventive protocols.

Croatia has around 3000 milk producers, and bovine dairy farms are mostly located in the continental part of Croatia, spread through 11 Croatian counties. In general, more than 100 microorganisms have been recognized worldwide as mastitis-causing agents. These are mostly bacteria. In 2015, Cvetnić et al. presented that 19.6% out of 3905 bovine milk samples collected on Croatian dairy farms were mastitis positive [7]. Deviation from basic animal hygiene and zootechnical measures, non-compliance with basic postulates for hygienic milking, and poor technical function of milking machines contribute to higher mastitis incidence. The most common causative agents of BM in Croatia were *Streptococcus uberis* (28.8%), *Staphylococcus aureus* (15.5%), *Streptococcus* spp. (13.8%), *Corynebacterium* spp. (8.6%), *Enterococcus* spp. (5.9%), *Trueperella pyogenes* (5.1%), *Staphylococcus* spp. (CNS) (4.8%), *Escherichia coli* (4.4%), pathogenic yeast (3.3%), *Streptococcus dysgalactiae* (2.1%), *Pasteurella* spp. (2%). Other bacterial agents, such as *Bacillus*, *Citobacter*, *Klebsiella*, *Proteus*, *Pseudomonas*, and *Serratia,* were isolated less often [7].

The expression of bacterial virulence and host immune factors can vary significantly depending on host factors, pathogen genera, species, strains, and course of infection [8]. Several reports have confirmed disparate immune responses to different pathogens and strains of bacteria during bovine mastitis [9,10]. Thus, we hypothesize that the milk proteome carries specific quantitative signatures of the host (bovine) proteins, discriminatory of *Staphylococcus* from *Streptococcus* mastitis-causing genera of bacteria. While several studies have reported the proteomic changes in bovine mastitic milk caused separately by members of the *Staphylococcus* [3,11,12] and the *Streptococcus* genera [2,6] to our knowledge, there has been no study directly comparing the pathogen-dependent host response to *Staphylococcus* and *Streptococcus* infection in bovine mastitis. Therefore, in the present study, we compared quantitative proteomic profiles of bovine milk samples naturally infected by *Staphylococcus* and *Streptococcus* species, independent of the mastitis level, and we targeted only bovine (host) proteins with significantly altered abundance between the two experimental groups. Proteomic profiles were generated by tandem-mass-tag (TMT)-based proteomics protocol and liquid chromatography-tandem mass spectrometry (LC-MS/MS), followed by statistical and bioinformatics analysis. Pathway enrichment analysis performed for altered proteins between two experimental groups potentially revealed different immune response pathways. Three proteins showed diagnostic potential for differentiating staphylococci and streptococci intramammary natural infection and inflammation.

## 2. Materials and Methods

### 2.1. Milk Samples

After a physical examination and before morning milking, milk samples (N = 118) were aseptically collected from mostly Holstein breed of dairy cattle on farms in Croatia (Supplementary Materials, File S1, Figure S1). Briefly, after disposing of the first three streams of milk, teats were thoroughly cleaned with water and disinfected with cotton wool wipes soaked in 70% (*v/v*) ethanol. Approximately 10 mL of udder secretion was collected per sample into a sterile polypropylene tube without the addition of preservatives and following the guidelines of the National Mastitis Council [13]. Samples were kept on ice and stored at 4 °C during transport and until microbiological analysis (Section 2.2). Afterward, samples were stored at −80 °C until mass spectrometry (MS)-based proteomics (Section 2.3). For MS-based proteomics, completely thawed milk samples were centrifuged (18,000× *g*, 30 min, 4 °C), and the middle portion (without cells and fat) was transferred to a clean polypropylene tube and analyzed.

### 2.2. Californian Mastitis Test and Microbiological Analysis

#### 2.2.1. Californian Mastitis Test (CMT)

CMT was performed in the laboratory using a mastitis test paddle and the reagent solution. Approximately 2 mL of milk sample was added to a cup of the paddle. An equal volume of the reagent solution was carefully squirted over milk to avoid bubble formation. The paddle is gently rotated in a horizontal position for 10 to 30 s. Positive reaction occurred and was graded during rotary motion. When the mixture of the milk sample and reagent solution remained liquid with no slime or gel formation, the reaction was classified as negative, and the tested quarter was defined as mastitis free. The reaction was mildly positive (+) if the mixture was slimy or gel-like, but by tipping the paddle back and forth, the mixture still covered the bottom of the cup. The reaction was graded as positive (++) if the mixture distinctly formed a gel. Mild (+) and positive (++) CMT tests represented cases of subclinical mastitis. A strongly positive reaction (+++) during CMT was recognized with the immediate formation of a gel. Swirling the cup moves the mixture toward the center of the cup, exposing the outer edges of the cup. A strongly positive reaction during CMT (+++) was classified as clinical mastitis.

#### 2.2.2. Identification of Mastitis-Causing Bacterial Pathogens in Milk

Microbiological examination of milk was carried out as described in the Laboratory handbook of bovine mastitis [13]. Briefly, 0.01 mL of milk sample was inoculated on nutrient agar with 5% of sheep blood and aesculin. Inoculated plates were incubated at 37 °C and checked for growing colonies after 18–24 h of incubation. Grown colonies were identified according to morphological and physiological properties (shape, color, diameter, surface, edges and hemolysis). Catalase-negative colonies resembling streptococci were submitted to the CAMP (Christie Atkinson Munch Peterson) test to distinguish *Streptococcus agalactiae* from other streptococci. Catalase-positive colonies resembling staphylococci were sub-cultivated on the Baird–Parker agar. CAMP test was performed using a *Staphylococcus aureus* strain causing incomplete hemolysis streaked along the diameter of a plate. Tested streptococcal strains were inoculated with the 90° angle against *S. aureus* and with a distance of 3 to 4 mm between. Inoculated plates were incubated at 37 °C overnight. The grown culture was checked for the formation of a wedge-shaped pattern radiating from the test organism near *S. aureus*. Non-agalactiae streptococci were additionally submitted to API 20 STREP test (Biomerieux, Marcy-l’Étoile, France) according to the manufacturer’s recommendation.

Selected colonies of staphylococci were inoculated on the Baird–Parker agar and incubated overnight at 37 °C. Grown colonies were checked for ability to coagulate rabbit plasma using the Bactident coagulase test (Merck, Rahway, NJ, USA) according to the manufacturer’s recommendations. Hence, several colonies were suspended in 0.5 mL of rabbit plasma and checked after 4 and 24 h. A positive reaction is manifested with gel formation in the bacterial suspension. Grown cultures were additionally tested using API STAPH (Biomerieux).

#### 2.2.3. Milk Sample Differentiation

Based on the results obtained from microbiological examination, milk samples were divided into experimental groups: *Staphylococcus* (N = 51) and *Streptococcus* (N = 67). Species present in the *Staphylococcus* group were *Staphylococcus aureus* (N = 32) and coagulase-negative staphylococci (CNS, N = 19), and in the *Streptococcus* group were *Streptococcus* spp. (N = 10), *Streptococcus uberis* (N = 25) and *S. dysgalactiae* (N = 32). A complete sample list is provided in Supplementary Materials File S2, Excel sheet: Legend for proteomics and samples.

### 2.3. MS-Based Quantitative Proteomics

#### 2.3.1. Sample Preparation for Bottom-Up TMT-Based Proteomics

To determine differences in relative protein quantities in milk between *Staphylococcus* and *Streptococcus* groups, the tandem-mass-tag (TMT)-MS-based proteomics approach was performed as previously described [1]. In brief, samples were centrifuged as explained in Section 2.1 and the middle portion of milk (further in the text referred to as the milk sample) was analyzed. Total protein concentration was determined with the Pierce BCA protein assay kit (Thermo Scientific, Rockford, IL, USA) by following the manufacturer’s protocol. Secondly, 35 µg of proteins per sample was transferred into both sample and internal standard tubes and were diluted with 0.1M triethyl ammonium bicarbonate (TEAB, Thermo Scientific, Rockford, IL, USA) to a final volume of 50 µL per sample. Therefore, an internal standard solution was made by blending 35 µg of proteins from all samples in the experiment. Consecutive bottom-up proteomics sample preparation steps were performed: (1) reduction (2.5 µL of 200 mM dithiothreitol (Sigma-Aldrich, St. Louis, MO, USA) solution at 55 °C for 60 min), (2) alkylation (2.5 µL of 375 mM (IAA, Sigma-Aldrich, St. Louis, MO, USA) solution at RT for 30 min), (3) protein precipitation with of 300 µL of ice-cold acetone (over-night at −20 °C, followed by centrifugation at 9000× *g*, 4 °C, 15 min), and (4) protein digestion with trypsin solution (Promega, Madison, WI, USA, 1 mg/mL, trypsin: protein ratio = 1:35, over-night at 37 °C). TMT 6-plex solutions (Thermo Scientific, Rockford, IL, USA) were freshly dissolved and used for the labeling of tryptic peptides by following the producer’s instructions. Briefly, 19 µL of the appropriate TMT label was added to every sample and internal standard. Incubation was performed at RT for 60 min and was stopped by the addition of 8 µL of 5% (*v/v*) hydroxylamine in 0.1M TEAB (Sigma-Aldrich, St. Lois, MO, USA). Finally, five appropriate TMT-labelled peptide samples were mixed with the internal standard into one TMT 6-plex, and these were divided into aliquots, which were vacuum dried and stored at −20 °C until nano-LC-MS/MS analysis for no more than two days. In total, 28 TMT 6-plex mixtures were prepared and analyzed (Supplementary Materials File S2, Excel sheet: Legend for proteomics and samples).

#### 2.3.2. Nano-LC-MS/MS Analysis and Data Processing

Vacuum-dried TMT-labelled tryptic peptides were dissolved in loading buffer (0.1% formic acid in 2% acetonitrile, *v/v*), while separation and detection of labeled peptides were performed on the UltiMate 3000 RSLCnano system (Thermo Fisher Scientific, Germering, Germany) coupled to the Q Exactive Plus Hybrid Quadrupole-Orbitrap mass spectrometer (Thermo Fisher Scientific, Bremen, Germany). The trap column (C18 PepMap100, 5 μm, 100A, 300 μm × 5 mm) and analytical column (PepMap™ RSLC C18, 50 cm × 75 μm) were from Thermo Fisher Scientific. The mass spectrometer was set to positive ion mode and Top8 data-dependent acquisition method. Parameters of liquid chromatography (LC), MS and MS/MS were adjusted, and raw data were processed as previously described [1]. Briefly, the linear LC gradient was used (see the gradient table provided in Supplementary Materials File S1, Table S1), MS spectra were collected from *m/z* 350.0 to *m/z* 1800.0, and the HCD fragmentation was used. Raw data were processed in the Proteome Discoverer (v.2.3., Thermo Fisher Scientific) with implemented SEQUEST algorithm. To identify bovine proteins present in milk, raw MS and MS/MS data were searched against *Bos taurus* FASTA files (downloaded from Uniprot/SwissProt on 21 January 2021, 120378 sequences). For the latter, two trypsin missed cleavages were allowed, and precursor (10 ppm) and fragment (0.02 Da) mass tolerance and protein modifications (carbamidomethyl (C) as static and oxidation (M) and TMT 6-plex (K, peptide N-terminus) as dynamic) were fixed. In order to accept protein identification, the following criteria had to be met: detection of at least two unique peptides per protein with a false detection rate (FDR) of less than 1%. The total peptide amount approach was chosen to normalize samples in one 6-plex, while an average of internal standard (TMT *m/z* 126 quan channel) was selected as a scaling mode between diverse 6-plex combinations. The proteomics data are deposited to the ProteomeXchange Consortium via the PRIDE partner repository [14,15] with the dataset identifier PXD041574.

#### 2.3.3. Statistics Analysis of Proteomics Results

To identify proteins with altered abundance between two experimental groups, statistical analysis (Mann–Whitney U test) was performed in R (v.4.1.2, accessed on 13 December 2021) using an in-house created script [16]. Only master proteins with significantly different abundances (*p* ˂ 0.05 and FDR ˂ 0.5) calculated in R were further analyzed.

#### 2.3.4. Bioinformatics Analysis of Proteomics Results

The list of quantified proteins was exported from the Proteome Discoverer, and for uncharacterized proteins (or proteins with no gene name), an additional search in the Basic Local Alignment Search Tool (BLAST) (v.2.9.0) (https://www.uniprot.org/blast (accessed on 19 January 2023)) [17,18] or via the NIH search tool provided by the National Centre for Biotechnology Information (NCBI) (https://www.ncbi.nlm.nih.gov/ (accessed on 19 January 2023)). The Protein Analysis Through Evolutionary Relationship (PANTHER) classification tool (v.17.0) (http://www.pantherdb.org/ (accessed on 13 February 2023)) [19] and STRING (v.11.5) (https://string-db.org/ (accessed on 13 February 2023)) [20] were used in the search for the protein’s class and functional analysis and creation of functional protein association networks. Therefore, gene names were inserted into PANTHER/STRING, and *Homo sapiens* were selected as the query organisms. The pathway over-representation analysis was performed in the REACTOME online tool (v. 83) (https://reactome.org/ (accessed on 15 February 2023)) using the curated database of pathways and reactions in *Homo sapiens* biology [21]. In calculations of pathways probability, a statistical (hypergeometric distribution) test was applied. The *p*-values were corrected for FDR calculated by Benjamini–Hochberg procedure. The pathway was considered significant when the corrected *p*-value ˂ 0.05 [22]. The receiver operating characteristic (ROC) curve analysis was performed in MedCalc (v.12.5.0.0, MedCalc, Ostend, Belgium).

## 3. Results

### 3.1. Milk Samples

**Figure 1:**
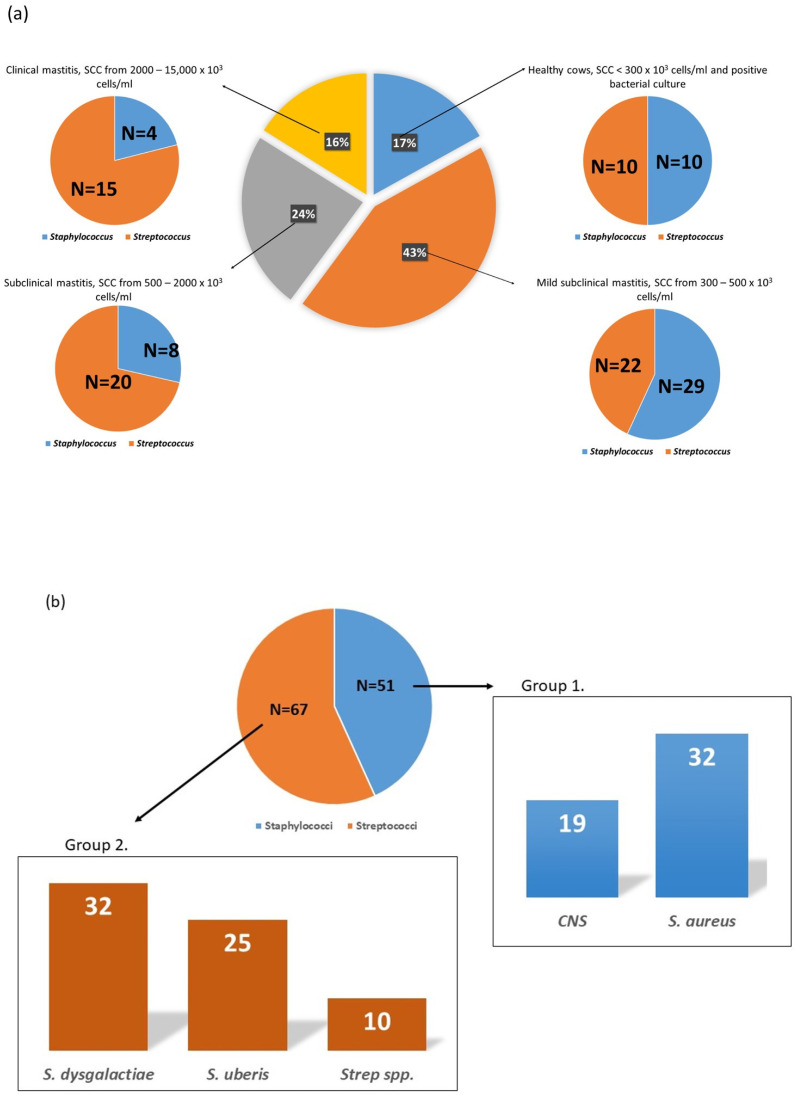
Distribution of mastitis cases in analyzed milk samples based on the results of the Californian mastitis test (**a**) and the number of milk samples with positive bacterial cultures: Group 1. *Staphylococcus* and Group 2. *Streptococcus* (**b**). The *Staphylococcus* group (Group 1.) was composed of milk samples positive for *Staphylococcus aureus* and coagulase-negative staphylococci (CNS), and the *Streptococcus* group (Group 2.) of milk samples classified as positive for *Streptococcus* spp, *Streptococcus uberis* and *S. dysgalactiae*.

A complete list of analyzed milk samples is provided in Supplementary Materials File S2, Excel sheet: Legend for proteomics and samples. CMT and microbiological examination were performed on the collected milk samples, and the results are presented in Figure 1.

### 3.2. Proteomics Results

Following TMT-based proteomics protocol and data analysis in Proteome Discoverer, 386 bovine proteins, of which 156 were master proteins, were quantified with ≥2 unique peptides, *p* < 0.05 and FDR < 0.001 (Supplementary Materials File S2, Excel sheet: Proteome Discoverer export). Of these proteins, 20 master proteins had different relative abundance (*p* < 0.05 and FDR < 0.5) between experimental groups and are presented in Table 1 together with selected statistical features, PANTHER protein classes (details are provided in Supplementary Materials File S2, Excel sheet: R export). Two proteins with the greatest change between experimental groups were odorant-binding protein-like (higher abundance in *Staphylococcus*) and fibrinogen beta chain (FGB) protein (higher abundance in *Streptococcus*) (Table 1).

**Figure 2:**
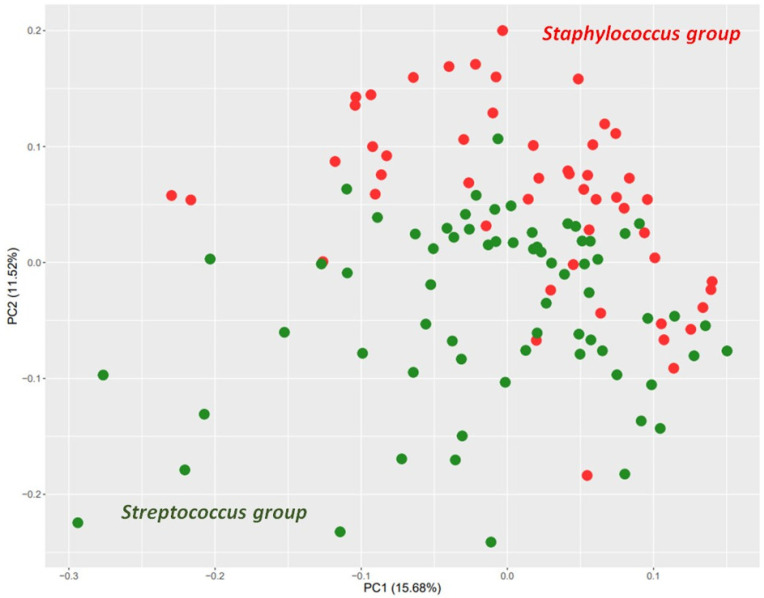
Principal component analysis (PCA) score plot representing the distinction of two experimental groups. The *Staphylococcus* group (N = 51) was composed of milk samples positive for *S. aureus* and coagulase-negative staphylococci (CNS), and the *Streptococcus* group (N = 67) of milk samples classified as positive for *Streptococcus* spp, *Streptococcus uberis* and *S. dysgalactiae*. Experimental groups were differentiated based on abundance ratios of proteins with statistically different abundances, shown in Table 1. PCA was performed in R (v.4.1.2).

The principal component analysis score plot (Figure 2) shows a distinction between experimental groups. Furthermore, three proteins showed the highest potential to differentiate between the *Streptococcus* and *Staphylococcus* groups with satisfactory accuracy (Table 2). These were protein kinase C-binding protein NELL2, thrombospondin-1, and complement factor I, which all had higher abundance in the *Staphylococcus* group and are classified as calcium-binding protein, cell adhesion molecule, and serine protease, respectively, in PANTHER (Table 1).

**Figure 3:**
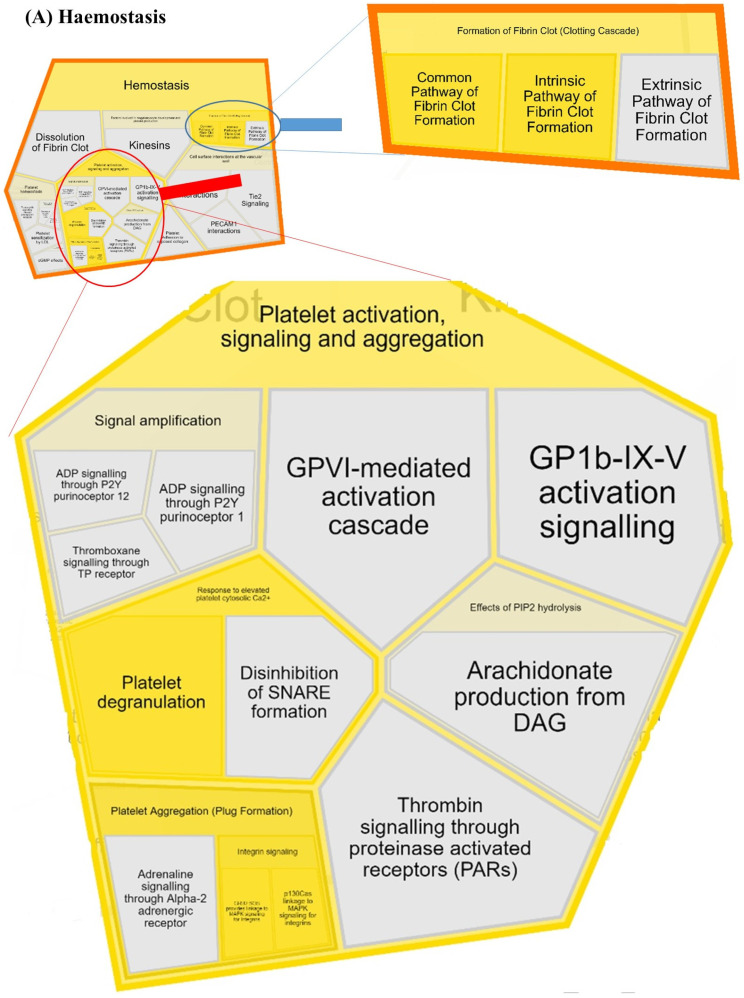

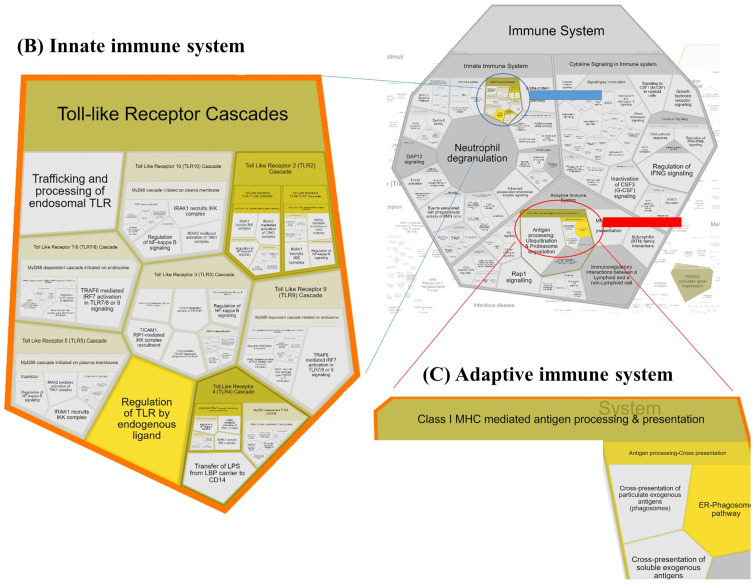

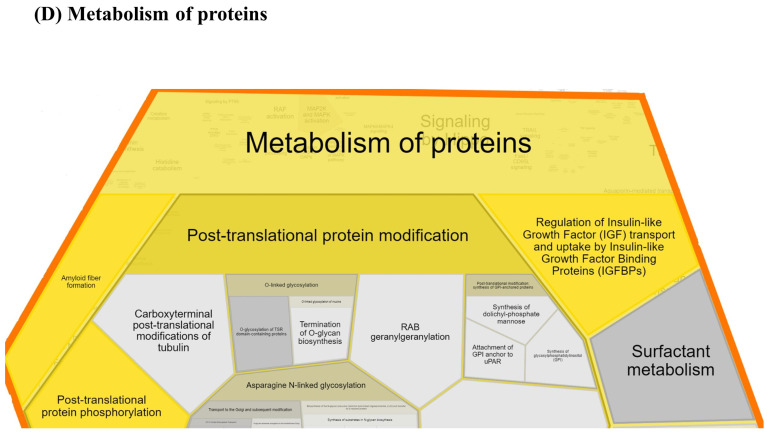

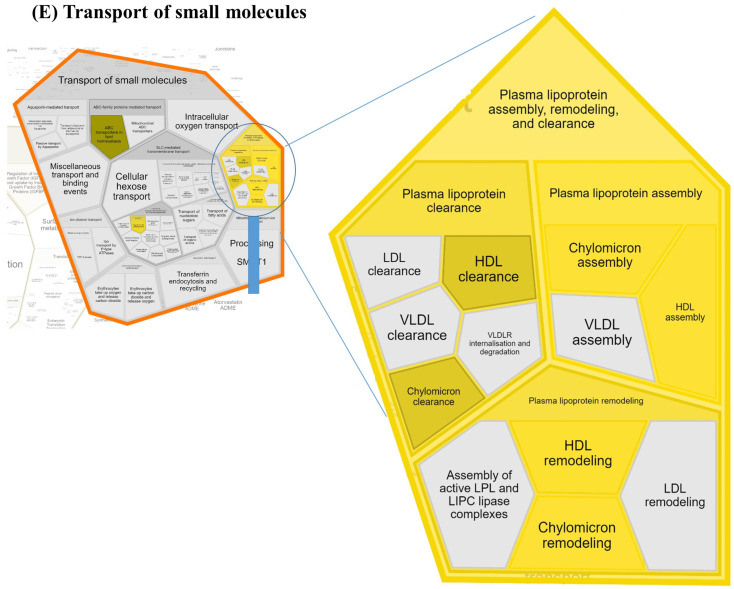

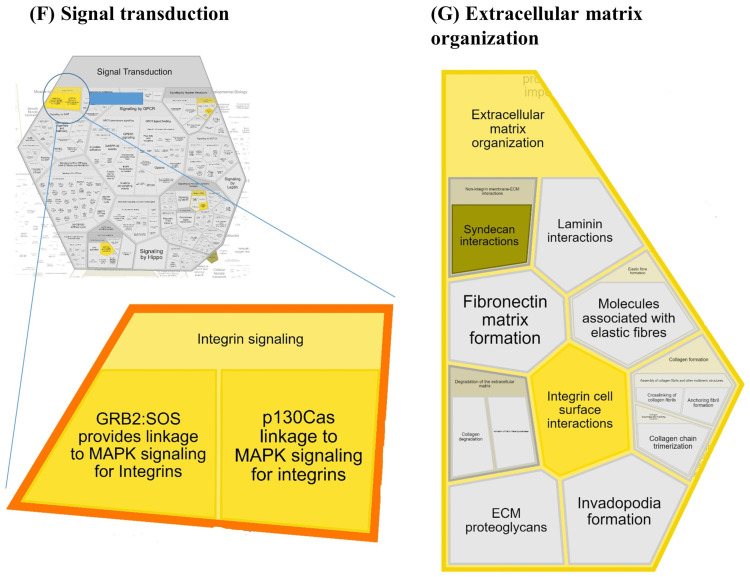
Reacfoam representation of enriched pathways: hemostasis (**A**), innate (**B**) and adaptive (**C**) immune system, metabolism of proteins (**D**), transport of small molecules (**E**), signal transduction (**F**), and extracellular matrix organization (**G**). Reackfoam was created in REACTOME online tool (v. 83) for differently abundant proteins between the *Staphylococcus* and *Streptococcus* groups (Table 1). Gene names MGC137014, MGC151921, LOC525947, and LOC100297192 were not found in Reactome.

**Figure 4:**
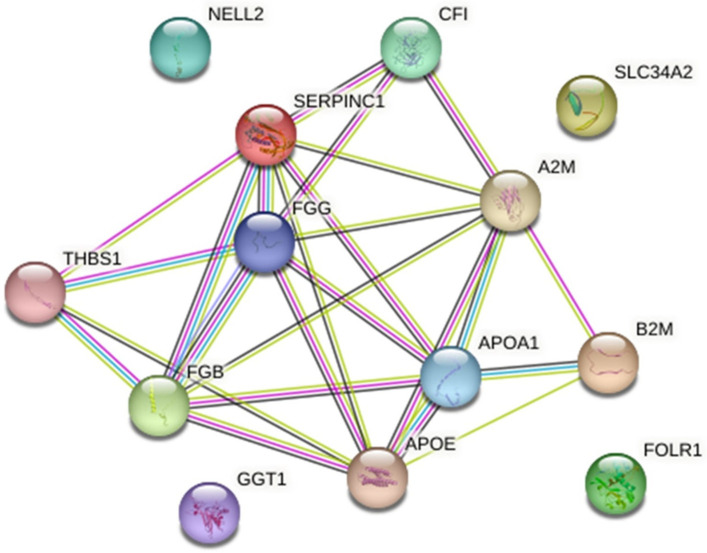
Potential protein/protein interaction networks created for differently abundant proteins between two experimental groups, the *Staphylococcus* and *Streptococcus.* Gene names are described in Table 1. Interaction network was created in STRING (v.11.5). In total, 13 proteins were found (gene names MGC151921, IGL, GLYCAM1, LOC100297192, LOC525947, and MGC137014 were not found). Network nodes represent all proteins (isoforms) produced by the single protein-coding locus. In the interaction network: colored nodes correspond to query proteins and the first shell of interactors, and white nodes to the second shell of interactors. Edges represent protein–protein associations, that is, proteins that jointly contribute to a shared function. Interactions (presented as colored lines) can be classified as known (from curated databases (turquoise) or determined experimentally (magenta)), predicted (gene neighborhood (green), gene fusions (red), gene co-occurrence (blue)) and others (text mining (yellow), co-expression (black) and protein homology (light blue)).

A Reactome analysis identified 15 out of 19 proteins in the Reactome database (MGC137014, MGC151921, LOC525947, and LOC100297192 were not found) mapped to 21 Reactome entities (Supplementary Materials, Figure S2A). In total, 186 pathways were hit with at least one gene name from the query list (Supplementary Materials, Figure S2B), and 25 pathways satisfied the cut-off criteria (*p* < 0.05 and FDR ˂ 0.05) (Table 3). Hemostasis (coagulation), innate and adaptive immune system, metabolism of proteins, transport of small molecules, signal transduction, and extracellular matrix organization pathways were enriched (Figure 3). STRING recognized 13 proteins (gene names MGC151921, IGL, GLYCAM1, LOC100297192, LOC525947, and MGC137014 were not found). Potential protein–protein interaction networks (Figure 4) and STRING functional enrichment (Figures S3 and S4) are presented.

## 4. Discussion

Morphologically, *Staphylococcus* and *Streptococcus* (although both Gram-positive cocci) differ in the arrangement of cells, cell division direction, as well as in their biochemical reaction to catalase, amongst others [23]. Significant proteome changes in milk have been shown to occur during mastitis [2,24]. These variations are a result of host-pathogen interactions during intramammary infection and inflammation, and proteins contributing to variations can be ascribed to both bacterial and host origin. Specifically, processes that contribute to milk proteomic changes are (1) the release of bacterial virulence proteins/factors and their corresponding biological activities, such as proteolysis of milk proteins, (2) the secretion of immune-responsive proteins from host tissue, (3) leakage of blood and mammary gland intracellular proteins into milk, and (4) altered mammary cellular metabolism resulting from the ensuing inflammation. Also, it has been established that the host responses to intramammary infection and inflammation fluctuate depending on virulence factors expressed by pathogens [25].

Twenty proteins had altered abundance between the two mastitis-causing bacteria genera examined (Table 1), which confirms our hypothesis that the milk proteome carries specific quantitative signatures of the host (bovine) proteins, discriminatory of *Staphylococcus* from *Streptococcus* mastitis-causing genera of bacteria. From the PANTHER/STRING gene ontology assessments, these proteins belong to diverse protein classes (Table 1), such as transfer/carrier proteins, cell adhesion and intracellular transfer molecules, serine proteases and protease inhibitors and major histocompatibility complex proteins. Differently abundant proteins are involved in different biological processes (Figure S3A) and molecular functions (Figure S3B): for instance, coagulation and response to wounding, platelet degranulation, regulation of response to stress and external stimulus, proteolysis, cell adhesion, transport, signal transduction, immune response, lipid metabolism, extracellular matrix organization, and others. Of these 20 proteins, only three proteins, all more abundant in the *Staphylococcus* group, showed diagnostic potential for discrimination of host (bovine) response to *Staphylococcus* contamination in milk (Table 2). These proteins were further discussed in Section 4.1 (below).

Most of the differentially abundant proteins between the two experimental groups had host defense functions (Table 3). This included proteins involved in (1) hemostasis, either through the formation of fibrin cloths or platelet activation, signaling and aggregation (Figure 3A), and (2) innate (Figure 3B) and adaptive (Figure 3C) immunity. This includes fibrinogen proteins (fibrinogen gamma-B chain and fibrinogen beta chain), complement proteins, and proteins with antimicrobial functions (complement factor I, fibrinogen gamma-B chain and fibrinogen beta chain protein) as well as proteins associated with the immune response to pathogens (immunoglobulin lambda locus, beta-2-microglobulin). Specifically, the toll-like receptor 2/4 (TLR2/4) cascade and its regulation by endogenous lipids (Figure 3B) and phagosome pathway, part of antigen processing-cross presentation (Figure 3C), were enriched pathways of immunity. The metabolism of proteins (Figure 3D, regulation of IGF transport and uptake by IGFBPs, amyloid fiber formations, and post-translational protein phosphorylation) and transport of small molecules (Figure 3E, plasma lipoprotein assembly, remodeling, and clearance) were enriched pathways. Furthermore, several proteins with different abundances were involved in signal transduction (Figure 3F, integrin signaling) and extracellular organization (Figure 3G, integrin cell surface interactions). Potential protein–protein interaction networks composed of 13 nodes (proteins) present proteins that jointly contribute to a shared function; however, four proteins (protein kinase C-binding protein NELL2, sodium-dependent phosphate transport protein 2B, glutathione hydrolase, and folate receptor alpha) were left out from network (Figure 4). Most presented interactions were based on experimental data and curated databases, some were, for example, predicted based on gene co-expression and text mining, and some interactions were based on both sources.

Overall, there were 11 proteins with higher abundance in *Staphylococcus* and nine in the *Streptococcus* group (Table 1). These changes in protein abundance point to different levels of innate/adaptive immune response dependent upon the pathogen present in the mammary gland (further discussed in Section 4.2 and Section 4.3)

### 4.1. Proteins with Altered Abundance between Staphylococcus and Streptococcus Experimental Groups That Show Diagnostic Potential

Prompt recognition of causative mastitis pathogens is important for control, prevention, and choice of treatment for BM [26]. Variations in the proteome of milk positive to mastitis-causing bacterial pathogens may be exploitable to not only gain a better understanding of the host responses but also for improved diagnostics of BM to the causative level.

#### 4.1.1. Protein Kinase C-Binding Protein NELL2 (NELL2)

NELL2 is a calcium-binding regulator of protein kinase C. Protein kinase C (PKC) is a member of protein kinase enzymes, cytoplasmic signal transducers, responsible for functional regulation and immune cell signaling through protein modification, specifically, phosphorylation (enriched REACTOME pathway, Table 3, Figure 3D) affecting cell proliferation and direct regulation of immune gene expression. PKC isoforms regulate both innate and adaptive immune systems, including toll-like receptor (TLR) signaling in the innate system (enriched REACTOME pathway, Table 3, Figure 3B). The activity of the PKC enzyme is regulated by increased concentration of metabolites and ions, for instance, Ca^2+^. Calcium is crucial for the activation of immune system cells, and subclinical hypocalcemia was identified as a factor predisposing Holstein cows to mastitis infections [27]. Moreover, calcium increase in milk is frequent after parturition, and large excretion of milk may result in calcium deficiency in blood and milk. Furthermore, calcium is also a blood-clotting agent and may increase in milk because of microhemorrhages in the mammary glands of cows, subsequently activating PKC. In the present study, the relative abundance of NELL2 protein, known as protein kinase C- and calcium-binding protein, was higher in the *Staphylococcus* group (Table 1). This may point to a potential role of NELL2 protein in the regulation of PKC activity/availability dependent upon calcium concentration in the milk, consequently resulting in the regulation of host immune pathogen-dependent response to the infection of mammary glands. On the other hand, PKC is also a major positive regulator of platelet granule secretion, integrin activation, and coagulation, which were all enriched biological pathways in the present study (Table 3, Figure 3A). In the study of Zhang et al. (2015) [28], NELL2 was identified and shown to increase in bovine milk with the progression of the lactation period.

#### 4.1.2. Thrombospondin-1 (THBS1)

THBS1 is a cell adhesion molecule, a regulator of Ca^2+^-dependent platelet (activation) degranulation and extracellular matrix organization dependent upon integrin receptors. As it is involved in the coagulation cascade, thrombospondin-1 is a glycoprotein found in connective tissues, plasma and alpha granules of platelets, involved in the regulation of attachment, proliferation and differentiation of cells, as was confirmed by PATHER (Table 1). It has been shown that bacteria cells bind this protein, and this helps them to get incorporated into the sub-epithelium of damaged tissues [29]. This protein was more abundant in the *Staphylococcus* group (Table 1), in which *S. aureus*, known for its adhesion and internalization within host cells, was one of identified mastitis-causing pathogens. Furthermore, the protein was recognized as an important factor of hemostasis recovery through platelet degranulation as a response to elevated Ca^2+^ (Table 3, Figure 3A) and extracellular matrix organization through interaction with integrin receptors (Table 3, Figure 3G). From previous proteomic studies, thrombospondin-1 was seen upregulated in bovine milk in response to a *Staphylococcus aureus* intramammary challenge [30] and also found in colostrum and mature milk [31].

#### 4.1.3. Complement Factor 1 (CF1)

CF1 is a serine protease that regulates immune response by controlling all complement pathways. The complement pathway is an important component of the innate immune system that supports antibody- and phagocytic-based clearance of pathogens by opsonization. A high abundance of complement factor 1 in the *Staphylococcus* group (Table 1) indicates inflamed mammary glands due to bacterial infection and early complement-mediated microbial lysis and activation of other immune components. Since immunoglobulins were also detected with altered abundance in the *Staphylococcus* group, CFI-mediated host response may be potentially classified as a classical complement pathway. The complement factor 1, which is a part of the complement pathway, is specifically involved in the cleavage and inactivation of C3b and Cab fragments in the complement cascade. High levels of this protein have been shown in bovine colostrum [32] and in both normal and mastitis milk [6]. Although the complement-mediated immune response may show effects on Gram-negative mastitis-causing pathogens, the bactericidal effect on Gram-positive, and especially *S. aureus* and biofilm-producing pathogens such as *S. uberis,* is still thought to be minor. Therefore, other immune factors seem to play a much greater role in *Staphylococcus* infection of mammary glands (Section 4.2).

### 4.2. Proteins and Associated Pathways More Abundant in the Staphylococcus Group

Immunoglobulin lambda locus (IGL@ protein), Ig-like domain-containing protein and immunoglobulin heavy constant mu protein had no available classification in PANTHER but along with antithrombin-III (classed as a protease inhibitor) and complement factor 1 (a serine protease), had higher abundance in the *Staphylococcus* group (Table 1). These proteins have mainly been reported in colostrum [31,32,33] but with minor representations in mature milk, as shown by the tendency to decrease as colostrum changes to mature milk in the study of Fahey et al. (2020) [33]. Similarly, these proteins did not have a very remarkable presence in mastitis milk (appearing in both infected and control samples) in the study of Smolenski et al. (2014) [6]. Next to involvement in the regulation of protein kinase C (Section 4.1.1) and classical complement pathway (Section 4.1.3), their immunologic roles, either in the conferment of adaptive immunity to the newborn or also their role during host mammary gland response to the pathogen invasion, is possible as these are primarily pre-existing factors in the healthy mammary gland environment [34]. The high abundance in the *Staphylococcus* group may further be a pointer of adaptive immune response to pathogens to which the host may have had previous exposure, such as ubiquitous *S. aureus* and CNS, to develop targeted immunity. In this way, immunoglobulins may act as surveillance immune factors in the early elimination of mastitis-causing pathogens. It has been established that immunoglobulins and acute-phase proteins increase in milk during infection [24].

Still, a number of the proteins, for instance, apolipoprotein E and serotransferrin-like protein, have been more strongly associated with colostrum [33] and, at the same time, mastitis [3], although they are also present in normal (uninfected) milk [33]. This points strongly to their direct role in immunity and inflammation, as has been suggested [35], even though there was also a report of apolipoprotein E decreasing during mastitis [1], which contradicts our findings in the present study. Serotransferrin-like protein has also been reported to increase in the course of mammary involution [36] and was seen during the resolution phase of an intramammary *S. uberis* challenge [2] and in the study of [6], possibly relating to its role in transport (Table 1).

The odorant-binding protein (OBP) has some structural similarities with the lipocalin family of proteins (inflammatory and detoxification processes) [37]. Through its transport/carrier functions (Table 1), it is possibly exploited by mastitis-causing pathogens to alter immune activation, avoid immune recognition, and thus promote the successful infection of the host. Such an interesting adaptation was previously reported for the fungus *Metarhizium anisopliae* infection in locusts [38]. The OBP has been variously identified as part of the milk proteome in colostrum [31,39] and in mature milk [38,40].

The glycosylation-dependent cell adhesion molecule 1 (GLYCAM-1), which is a hormonally regulated endothelial adhesion glycoprotein expressed in lactating mammary glands, has been reported by several authors in colostrum [33] and mature milk [6,24,39], also known to be involved in the immune response. Although with no classification on PANTHER (Table 1), the abundance of GLYCAM-1 significantly decreased during the course of lactation progression [28] and in subclinical *S. uberis* mastitis milk compared to controls [6]. There was no appreciable change in this protein during the peak of an *S. uberis* mastitis, although peptides arising from the proteolysis of this protein have been shown to be mainly decreased in milk at the peak of an *S. uberis* mastitis [2]. Similarly, peptides originating from GLYCAM-1 were reduced in mastitis caused by CNS compared to controls, as reported by Addis et al., 2020 [41]. This contradictory behavior could agree with the finding that the protein has no diagnostic potential to differentiate the two groups in the present study (Table 2).

### 4.3. Proteins and Associated Pathways More Abundant in the Streptococcus Group

None of the proteins in this group showed potential diagnostic value in differentiating the two experimental groups (Table 2). Beta-2-microglobulin (which is the beta chain of a major histocompatibility complex (MHC) class I molecule) is involved in host immune responses and abundantly expressed in colostrum [32,33]. Its higher abundance in the *Streptococcus* group could indicate the greater involvement of the MHC in response to this group of pathogens. Interestingly, beta-2-microglobulin was shown to increase by several folds in *S. aureus* mastitis [3] but decreased significantly in an experimental *S. uberis* intramammary challenge compared to controls [6]. Although a comparison of the abundance of the proteins in these studies and ours cannot be made, these reports contrast our findings of the protein being higher in *Streptococcus* than in *Staphylococcus*.

Fibrinogen abundance was higher in the *Streptococcus* group (Table 1), indicating a potentially higher level of tissue damage and/or different pathways of pathogen intrusion compared to *Staphylococcus*. Specifically, the degradation of the extracellular matrix due to plasmin activation facilitates *S. uberis* invasion [26]. In line with this, proteins with higher abundance in the *Streptococcus* group (Table 1) were frequently enriched in hemostasis recovery, specifically, (1) formation of fibrin cloth and (2) platelet activation, signaling and aggregation (Table 3, Figure 3A) as well as extracellular matrix organization through integrin surface interactions (probably with fibrinogen) (Table 3, Figure 3G). A higher abundance of alpha-2-macroglobulin, fibrinogen and IgG proteins in the *Streptococcus* group may act negatively on the host response to the infection by *S. dysgalactiae*. As previously reported, binding of these proteins to pathogen cells may lead to decreased opsonization and reduced phagocytosis [26].

Integrin signaling is linked to the mitogen-activated protein kinase (MAPK) pathway (Table 3) by recruiting two different activation complexes (Figure 3F). In addition, fibrinogen gamma-B chain and fibrinogen beta chain proteins were enriched in a number of biological pathways (Table 3), including a primary line of defense, that is, the regulation of toll-like receptor (TLR) family of pattern recognition receptors (Table 3, Figure 3B) important for bacterial intramammary infections.

Sodium-dependent phosphate transport protein 2B has formerly been reported as a regular constituent of normal milk with less abundance in colostrum [42] and intramammary infection and inflammation [1,31]. Indeed, the concentration of this protein has been shown to decrease mastitis in Simmental cows [1], while peptides originating from the proteolysis of this protein have been shown to increase in cow milk during CNS mastitis [41].

### 4.4. Study Limitations and Future Research Plans

In the present study, the clinical/subclinical form of the disease, that is, the level of somatic cell counts (SCC), was not taken into consideration during the assignment of experimental groups and in discriminating protein abundance changes. This lack of subdivision of experimental groups dependent upon mastitis level might be considered a study limitation, and it is in our interest to upgrade the results with future research. Separating those groups might put some of the factors we have determined in a unique position to be predictive of, for instance, early-grade (subclinical) mastitis caused by specific streptococci. The next step of investigation might be the detection of proteome changes between two experimental groups (*Staphylococcus* and *Streptococcus*) dependent upon mastitis level. Specifically, milk samples collected from dairy cows diagnosed with similar clinical forms of mastitis caused by the same bacterial pathogen would form one experimental group. Cognate studies targeting comparisons of other common mastitis-causing genera of bacteria could prove beneficial in further unraveling the enigma of host-pathogen interactions during specific-pathogen intramammary infection and inflammation.

It has been said that the quantitative proteome in samples could be useful in evaluating the extent of host response, progress of disease and effect of treatment [24]. With the continuous upgrade of -*omics* techniques, protocols and analytical instrumentation, specifically for mass spectrometry-based proteomics in the last few decades, we have seen the possibility of quantitative differentiation of the proteome between biological matrices at a large scale. This approach could prove valuable in the early diagnosis of mastitis and differentiation of mastitis-causing etiologies. We recognize that mass spectrometry is still generally considered expensive instrumentation, which involves requirements for highly skilled operators and data analysts. Invariably, mass spectrometry-based proteomics is a high-throughput technique, which is highly sensitive and specific, and when operated with care, provides results of increased repeatability and accuracy. Therefore, we propose a mass spectrometry-based study approach in the marker discovery and validation phases, while other protein-based technologies that may arise on MS results, such as ELISA, could be more accepted in the daily laboratory routine.

## 5. Conclusions

The results of the present study, which showed quantitative differences in the milk proteome in *Streptococcus* versus *Staphylococcus* infection, could potentially offer means for promptly differentiating these infections. Thus, the use of these quantitative signatures, as preliminarily found here, to show diagnostic potentials, for example, protein kinase C-binding protein NELL2 and others, may become profitable and applicable for differentiating between these two predominant pathogen species that frequently present with environmental (*Streptococcus*) and contagious (*Staphylococcus*) transmission dynamics.

## Figures and Tables

**Table 1:** Bovine proteins with altered relative abundance (*p* < 0.05, FDR < 0.5) between experimental groups *Staphylococcus* (N = 51) and *Streptococcus* (N = 67) with their respective PANTHER protein classes. Statistical analysis was performed in R (v.4.1.2) and gene ontology in PANTHER (v. 17.0). Eleven proteins were more abundant in the *Staphylococcus* and nine in the *Streptococcus* group.

Uniprot Accession	Description/BLAST Search	Gene Name	*p*-Value	FDR	FC *	log2FC	PANTHER Protein Class (Class ID)
Proteins more abundant in the *Staphylococcus*-positive milk samples							
Q0IIA2	Odorant-binding protein-like	MGC151921	0.006	0.192	0.774	−0.370	transfer/carrier protein (PC00219)
Q3T101	IGL@ protein	IGL	0.017	0.365	0.815	−0.295	no class available in PANTHER
A0A140T881	Apolipoprotein E	APOE	0.045	0.430	0.844	−0.245	apolipoprotein (PC00052)
P80195	Glycosylation-dependent cell adhesion molecule 1	GLYCAM1	0.035	0.430	0.868	−0.204	no class available in PANTHER
G3N2D7	Uncharacterized protein/Ig-like domain-containing protein	LOC100297192	0.002	0.106	0.874	−0.194	not found in PANTHER
A6QR11	Protein kinase C-binding protein NELL2	NELL2	0.014	0.338	0.874	−0.194	calcium-binding protein (PC00060)
Q2HJF0	Serotransferrin-like	LOC525947	0.047	0.430	0.877	−0.189	transfer/carrier protein (PC00219)
F1N3A1	Thrombospondin-1	THBS1	0.009	0.220	0.887	−0.172	cell adhesion molecule (PC00069)
G5E513	Uncharacterized protein/Immunoglobulin heavy constant mu	not available	0.037	0.430	0.905	−0.145	not available
Q32PI4	Complement factor I	CFI	0.001	0.105	0.905	−0.144	serine protease (PC00203)
F1MSZ6	Antithrombin-III	SERPINC1	0.003	0.123	0.919	−0.123	protease inhibitor (PC00191)
Proteins more abundant in the *Streptococcus*-positive milk samples							
P01888	Beta-2-microglobulin	B2M	0.050	0.430	1.094	0.130	major histocompatibility complex protein (PC00149)
P02702	Folate receptor alpha	FOLR1	0.021	0.402	1.100	0.137	membrane trafficking regulatory protein (PC00151)
F1N6D4	Sodium-dependent phosphate transport protein 2B	SLC34A2	0.024	0.421	1.112	0.153	secondary carrier transporter (PC00258)
Q7SIH1	Alpha-2-macroglobulin	A2M	0.029	0.430	1.114	0.156	protease inhibitor (PC00191)
G3N2D8	Uncharacterized protein/Glutathione hydrolase	GGT1	0.006	0.192	1.128	0.174	protease (PC00190)
P15497	Apolipoprotein A-I	APOA1	0.038	0.430	1.153	0.205	apolipoprotein (PC00052)
Q2KIT0	Protein HP-20 homolog	MGC137014	0.002	0.106	1.180	0.239	not available in PANTHER
Q3SZZ9	FGG protein/Fibrinogen gamma-B chain	FGG	0.002	0.106	1.186	0.246	intercellular signal molecule (PC00207)
A6QPX7	FGB protein (Fragment)/Fibrinogen beta chain protein	FGB	0.001	0.105	1.277	0.353	intercellular signal molecule (PC00207)

* FC = Fold change; IGL = Immunoglobulin lambda locus; NELLE = neural epidermal growth factor -like-like 2.

**Table 2:** Results of the ROC analysis indicate the diagnostic potential of bovine proteins with altered relative abundance (*p* ˂ 0.05, FDR ˂ 0.5) between experimental groups *Staphylococcus* (N = 51) and *Streptococcus* (N = 67).

Protein	Uniprot Accession	AUC *	Sensitivity (%) **	Specificity (%) ***	Positive Likelihood Ratio #	Negative Likelihood Ratio ##
Proteins more abundant in the *Staphylococcus*-positive milk samples						
Apolipoprotein E	A0A140T881	0.608 (0.514–0.696)	45 (31–60)	76 (64–86)	1.9 (1.1–3.2)	0.7 (0.5–1.0)
Antithrombin-III	F1MSZ6	0.657 (0.559–0.747)	63 (48–77)	64 (51–76)	1.8 (1.2–2.7)	0.6 (0.4–0.9)
Protein kinase C-binding protein NELL2	A6QR11	0.658 (0.546–0.759)	47 (30–65)	*94 (83–99)*	*7.7 (2.4–24.3)*	0.6 (0.4–0.8)
Thrombospondin-1	F1N3A1	0.652 (0.557–0.739)	*82 (69–96)*	50 (37–63)	1.6 (1.2–2.2)	0.4 (0.2–0.7)
Glycosylation-dependent cell adhesion molecule 1	P80195	0.613 (0.519–0.702)	78 (65–89)	43 (31–56)	1.4 (1.1–1.8)	0.5 (0.3–0.9)
Odorant-binding protein-like	Q0IIA2	0.645 (0.552–0.731)	67 (52–79)	61 (48–72)	1.7 (1.2–2.5)	0.5 (0.4–0.8)
Serotransferrin-like	Q2HJF0	0.662 (0.533–0.775)	58 (37–77)	71 (54–85)	2.0 (1.1–3.6)	0.6 (0.4–1.0)
Complement factor I	Q32PI4	0.692 (0.587–0.783)	65 (49–79)	*74 (60–85)*	*2.5 (1.5–4.2)*	*0.5 (0.3–0.7)*
IGL@ protein	Q3T101	0.629 (0.535–0.716)	63 (48–76)	62 (48–73)	1.6 (1.1–2.3)	0.6 (0.4–0.9)
Ig-like domain-containing protein	G3N2D7	0.678 (0.585–0.761)	57 (42–71)	79 (67–88)	2.7 (1.6–4.6)	0.6 (0.4–0.8)
Immunoglobulin heavy constant mu	G5E513	0.574 (0.476–0.667)	51 (37–65)	64 (50–76)	1.4 (0.9–2.2)	0.8 (0.5–1.1)
Proteins more abundant in the *Streptococcus*-positive milk samples						
Beta-2-microglobulin	P01888	0.61 (0.513–0.702)	69 (54–81)	54 (40–67)	1.5 (1.1–2.1)	0.6 (0.4–0.9)
Fibrinogen beta chain protein	A6QPX7	0.670 (0.578–0.754)	55 (43–67)	74 (60–86)	2.2 (1.3–3.6)	0.6 (0.4–0.8)
Sodium-dependent phosphate transport protein 2B	F1N6D4	0.622 (0.528–0.709)	60 (47–71)	65 (50–78)	1.7 (1.1–3.6)	0.6 (0.4–0.9)
Folate receptor alpha	P02702	0.630 (0.537–0.717)	67 (55–78)	57 (42–71)	1.6 (1.1–2.2)	0.6 (0.4–0.9)
Apolipoprotein A-I	P15497	0.607 (0.513–0.696)	85 (74–93)	35 (22–50)	1.3 (1.0–1.6)	0.4 (0.2–0.8)
Protein HP-20 homolog	Q2KIT0	0.701 (0.572–0.810)	76 (59–89)	62 (42–79)	2.0 (1.2–3.3)	0.4 (0.2–0.7)
Fibrinogen gamma-B chain	Q3SZZ9	0.658 (0.564–0.742)	76 (64–86)	57 (42–71)	1.8 (1.3–2.5)	0.4 (0.3–0.7)
Alpha-2-macroglobulin	Q7SIH1	0.614 (0.520–0.702)	52 (40–65)	73 (58–84)	1.9 (1.2–3.1)	0.7 (0.5–0.9)
Glutathione hydrolase	G3N2D8	0.647 (0.554–0.733)	51 (38–63)	76 (62–87)	2.1 (1.2–3.7)	0.6 (0.5–0.9)

* Area under the curve (AUC) with 95% confidence interval. ** Sensitivity with 95% confidence interval. *** Specificity with 95% confidence interval. # Positive likelihood ratio 95% confidence interval. ## Negative likelihood ratio 95% confidence interval.

**Table 3:** The pathway over-representation analysis results for bovine proteins differentially abundant in milk samples distributed between two experimental groups, *Staphylococcus* (N = 51) and *Streptococcus* (N = 67). Pathway enrichment was performed in REACTOME (v. 83), with gene names shown in Table 1. Pathway was considered significant when the corrected *p*-value ˂ 0.05. Gene hits shown in bold letters were more abundant in the *Streptococcus* group.

Pathway Name (Identifier)	Entities				Reactions
	*p*-Value	FDR *	Found/Total Number of Genes in the Pathway	Gene Hits	Found/Total Number of Reactions
Formation of Fibrin Clot/Clotting Cascade (R-HSA-140877)	7.65 × 10^−7^	1.36 × 10^−4^	4/43	**A2M**, **FGG**, **FGB**, SERPINC1	12/61
Platelet degranulation (R-HSA-114608)	3.09 × 10^−6^	2.30 × 10^−4^	5/141	**A2M**, **FGG**, **APOA1**, THBS1, **FGB**	2/11
Response to elevated platelet cytosolic Ca^2+^ (R-HSA-76005)	3.9 × 10^−6^	2.30 × 10^−4^	5/148	**A2M**, **FGG**, **APOA1**, THBS1, **FGB**	2/14
Common pathway of fibrin clot formation (R-HSA-140875)	9.88 × 10^−6^	4.35 × 10^−4^	3/25	**FGB**, **FGG**, SERPINC1	9/29
Plasma lipoprotein assembly (R-HSA-8963898)	1.7 × 10^−5^	5.94 × 10^−4^	3/30	**A2M**, **APOA1**, APOE	9/19
Post-translational protein phosphorylation (R-HSA-8957275)	2.94 × 10^−5^	8.51 × 10^−4^	4/109	**APOA1**, **FGG**, APOE, SERPINC1	1/1
Regulation of IGF transport and uptake by IGFBPs (R-HSA-381426)	5.30 × 10^−5^	0.001	4/127	**APOA1**, **FGG**, APOE, SERPINC1	1/14
Platelet activation, signaling and aggregation (R-HSA-76002)	1.01 × 10^−4^	0.002	5/293	**A2M**, **FGG**, **APOA1**, THBS1, **FGB**	17/117
Chylomicron assembly (R-HSA-8963888)	2.5 × 10^−4^	0.005	2/14	**APOA1**, APOE	3/5
Chylomicron remodeling (R-HSA-8963901)	3.67 × 10^−4^	0.005	2/17	**APOA1**, APOE	3/3
Integrin cell surface interactions (R-HSA-216083)	3.77 × 10^−4^	0.005	3/86	**FGB**, **FGG**, THBS1	5/55
HDL assembly (R-HSA-8963896)	4.11 × 10^−4^	0.005	2/18	**A2M**, **APOA1**	6/9
Amyloid fiber formation (R-HSA-977225)	4.16 × 10^−4^	0.005	3/89	**APOA1**, APOE, **B2M**	2/33
GRB2:SOS provides linkage to MAPK signaling formation (R-HSA-354194)	5.06 × 10^−4^	0.006	2/20	**FGB**, **FGG**	2/2
P130Cas linkage to MAPK signaling integrins (R-HSA-372708)	6.11 × 10^−4^	0.006	2/22	**FGB**, **FGG**	3/3
Plasma lipoprotein assembly, remodeling, and clearance (R-HSA-174824)	6.18 × 10^−4^	0.006	3/102	**A2M**, **APOA1**, APOAE	31/86
HDL remodeling (R-HSA-8964058)	7.26 × 10^−4^	0.006	2/24	**APOA1**, APOE	10/13
MyD88 deficiency (TLR2/4) (R-HSA-5602498)	8.51 × 10-4	0.006	2/26	**FGB**, **FGG**	2/2
Intrinsic pathway of fibrin clot formation (R-HSA-140837)	8.51 × 10^−4^	0.006	2/26	**A2M**, SERPINC1	3/24
IRAK4 deficiency (TLR2/4) (R-HSA-5603041)	9.16 × 10^−4^	0.006	2/27	**FGB**, **FGG**	2/2
Glutathione synthesis and recycling (R-HSA-174403)	9.16 × 10^−4^	0.006	2/27	**GGT1**	1/9
Haemostasis (R-HSA-109582)	0.002	0.011	6/803	**A2M**, **FGG**, **APOA1**, SERPINC1, **FGB**, THBS1	29/338
Regulation of TLR by endogenous ligand (R-HSA-5686938)	0.002	0.011	2/36	**FGB**, **FGG**	1/12
Integrin signaling (R-HSA-354192)	0.002	0.011	2/39	**FGB**, **FGG**	15/24
Extracellular matrix organization (R-HSA-1474244)	0.002	0.011	4/328	**A2M**, **FGG**, **FGB**, THBS1	7/319

* FDR = false discovery rate; IGF = insulin-like growth factor; IGFBPs = Insulin-like growth factor binding proteins; HDL = high-density lipoprotein; Grb2 = growth factor receptor-bound protein 2; Sos = son of sevenless homologue protein; MAPK = mitogen-activated protein kinase; TLR2 = Toll-like receptor.

## Data Availability

The mass spectrometry proteomics data have been deposited to the ProteomeXchange Consortium via the PRIDE partner repository with the dataset identifier PXD041574. The preliminary data, without detailed bioinformatics analysis and interpretation, were presented as a poster with the title “Analysis of milk proteome in differentiating between Staphylococci and Streptococci as the causative microbes of mastitis in dairy cows” during the 7th Croatian Congress of Microbiology with International Participation, held from 24–27 May 2022 in Sv. Martin na Muri, Croatia. Only the first draft of the abstract is available in the Book of Abstracts.

## Supplementary Materials

The following supporting information can be downloaded at: https://www.mdpi.com/article/10.3390/ani13111829/s1; Supplementary Materials File S1: Figure S1: Distribution of Croatian dairy farms where milk samples were collected; Table S1. Liquid chromatography-mass spectrometry mobile phase gradient; Figure S2. Reactome analysis. A list of the input identifiers that have been found or mapped to an equivalent element in Reactome, classified by resource (A). Reacfoam representation of pathways hit with at least one gene name from the query list (B); Figure S3. STRING functional analysis in the analyzed network created for differently abundant proteins between two experimental groups, Staphylococci and Streptococci; Figure S4. STRING functional analysis in the analyzed network created for differently abundant proteins between two experimental groups, Staphylococci and Streptococci; Supplementary Materials File S2: Excel sheet 1. Proteome Discoverer export; Excell sheet 2. Legend for proteomics and samples; Excel sheet 3. R export.
