# Supplementary material for: Distinguishing Natural Infections of the Bovine Mammary Gland by Staphylococcus from Streptococcus spp. Using Quantitative Milk Proteomics
Source: Animals (Basel). 2023 May 31;13(11):1829. doi: 10.3390/ani13111829 (PMC10252062; doi:10.3390/ani13111829)
Supplement: Supplementary file 1 [file animals-13-01829-s001.zip › Supplementary material_File S1.pdf]

Supplementary material A

Distinguishing natural infections of the bovine mammary gland by *Staphylococcus* from *Streptococcus* spp. using quantitative milk proteomics

Dina Rešetar Maslov [1,* †], Funmilola Clara Thomas [1,*], Anđelo Beletić [1], Josipa Kuleš [2], Ivana Rubić [1], Miroslav Benić [3], Goran Bačić [4], Nino Maćešić [4], Vida Eraghi [1], Vladimir Farkaš[1], Tihana Lenac Roviš [5], Berislav Lisinić [5], Damir Žubčić [6], Dalibor Potočnjak [6], Vladimir Mrljak [1,6]

[1] Laboratory of proteomics, Internal Diseases Clinic, Faculty of Veterinary Medicine, University of Zagreb, Heinzelova street 55, 10000 Zagreb, Croatia

[2] Department of Chemistry and Biochemistry, Faculty of Veterinary Medicine, University of Zagreb, Heinzelova street 55, 10000 Zagreb, Croatia

[3] Department of bacteriology and parasitology, Croatian Veterinary Institute, Savska cesta, 143, 10000 Zagreb, Croatia

[4] Reproduction and Obstetrics Clinic, Faculty of Veterinary Medicine, University of Zagreb, Heinzelova street 55, 10000 Zagreb, Croatia

[5] Center for proteomics University of Rijeka, Faculty of medicine, Brace Branchetta 20

51000 Rijeka, Croatia

[6] Internal Diseases Clinic, Faculty of Veterinary Medicine, University of Zagreb, Heinzelova street 55, 10000 Zagreb, Croatia

* These authors contributed equally.

† Corresponding author: drmaslov@vef.hr

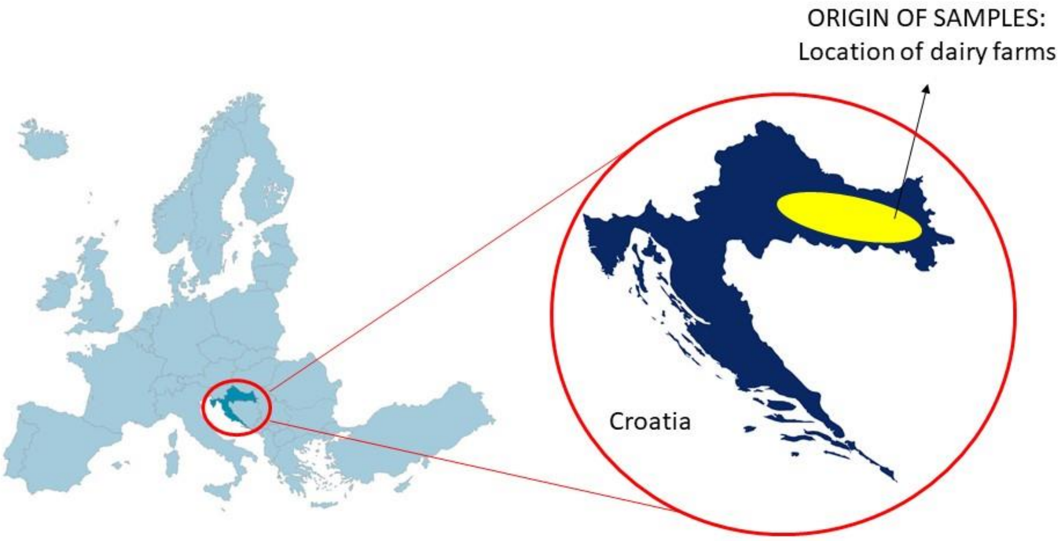

Figure S1. Distribution of Croatian dairy farms where milk samples were collected. This included six Croatia counties: Osječko-Baranjska, Bjelovarsko-Bilogorska, Križevačko-Koprivnička, Krapinsko-Zagorska, Sisačko-Moslavačka i Zagrebačka county.

Table S1. Liquid chromatography – mass spectrometry mobile phase gradient.

Time (min)	Flow (nL/min)	% of mobile phase B#
0 - 120	300	5-45%
120-122	300	45-90%
122-124	300	80%
124-144	300	5%

0.1% formic acid (vol/vol) in 80% acetonitrile (vol/vol) diluted in ultrapure water

(A)

Input	UniProt Id	Input	UniProt Id	Input	UniProt Id
A2M	P01023	APOA1	P02647	APOE	P02649
B2M	P61769	CFI	P05156	FGB	P02675
FGG	P02679	FOLR1	P14207, P15328	GGT1	A6NGU5, P19440
GLYCAM1	Q8IVK1	IGL	P17677	NELL2	Q99435
SERPINC1	P01008	SLC34A2	O95436	THBS1	P07996

Input	Ensembl Id	Input	Ensembl Id
APOA1	ENSG00000118137	APOE	ENSG00000130203
B2M	ENSG00000166710	THBS1	ENSG00000137801

(B)

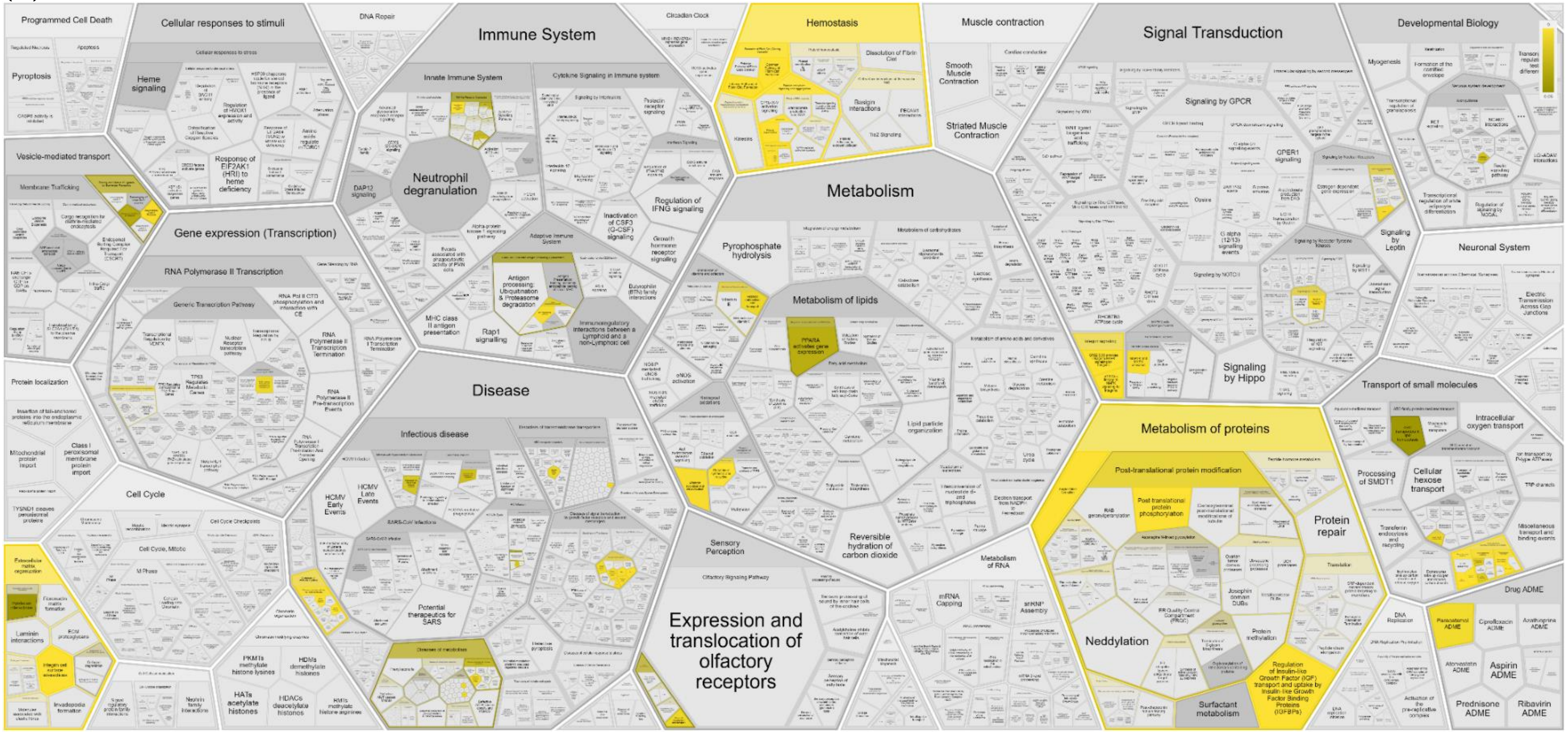

Figure S2. Reactome analysis. A list of the input identifiers that have been found or mapped to an equivalent element in Reactome, classified by resource (A). Reacfoam representation of pathways hit with at least one gene name from the query list (B). Pathways (without cut-off) that were enriched are coloured yellow. Reacfoam was exported from Reactome software following pathway enrichment analysis of serum proteins differentially abundant between experimental groups.

(A)

GO-term	description	count in network	strength	false discovery rate
	**Biological Process (Gene Ontology)**			
GO:0030193	Regulation of blood coagulation	5 of 80	1.97	2.20e-05
GO:0002576	Platelet degranulation	5 of 129	1.77	5.51e-05
GO:0080134	Regulation of response to stress	9 of 1437	0.97	7.44e-05
GO:0032101	Regulation of response to external stimulus	8 of 1013	1.08	9.95e-05
GO:0032102	Negative regulation of response to external stimulus	6 of 367	1.39	0.00010
GO:0016192	Vesicle-mediated transport	9 of 1805	0.88	0.00032
GO:0050878	Regulation of body fluid levels	6 of 509	1.25	0.00055
GO:0009611	Response to wounding	6 of 532	1.23	0.00065
GO:0034114	Regulation of heterotypic cell-cell adhesion	3 of 25	2.26	0.00073
GO:0072378	Blood coagulation, fibrin clot formation	3 of 26	2.24	0.00076
GO:1901564	Organonitrogen compound metabolic process	12 of 5244	0.54	0.0011
GO:0048583	Regulation of response to stimulus	11 of 4114	0.6	0.0014
GO:1900026	Positive regulation of substrate adhesion-dependent cell spr...	3 of 38	2.07	0.0015
GO:0046903	Secretion	7 of 1097	0.98	0.0015
GO:0009605	Response to external stimulus	9 of 2310	0.77	0.0015
GO:0019538	Protein metabolic process	11 of 4251	0.59	0.0016
GO:0045055	Regulated exocytosis	6 of 697	1.11	0.0017
GO:0006810	Transport	11 of 4353	0.58	0.0018
GO:0031638	Zymogen activation	3 of 44	2.01	0.0020
GO:1902995	Positive regulation of phospholipid efflux	2 of 3	3.0	0.0021
GO:0030195	Negative regulation of blood coagulation	3 of 47	1.98	0.0021
GO:2000351	Regulation of endothelial cell apoptotic process	3 of 50	1.96	0.0023
GO:0006897	Endocytosis	5 of 433	1.24	0.0023
GO:0006950	Response to stress	10 of 3485	0.64	0.0023
GO:1902041	Regulation of extrinsic apoptotic signaling pathway via deat...	3 of 59	1.88	0.0030
GO:0055081	Anion homeostasis	3 of 61	1.87	0.0032
GO:0010810	Regulation of cell-substrate adhesion	4 of 212	1.45	0.0032
GO:0032489	Regulation of cdc42 protein signal transduction	2 of 7	2.63	0.0047
GO:0060627	Regulation of vesicle-mediated transport	5 of 550	1.14	0.0060
GO:0002682	Regulation of immune system process	7 of 1514	0.84	0.0060
GO:0010873	Positive regulation of cholesterol esterification	2 of 9	2.52	0.0064
GO:0034371	Chylomicron remodeling	2 of 9	2.52	0.0064
GO:0034372	Very-low-density lipoprotein particle remodeling	2 of 9	2.52	0.0064
GO:0065008	Regulation of biological quality	10 of 4042	0.57	0.0064
GO:0034378	Chylomicron assembly	2 of 10	2.48	0.0068
GO:0034384	High-density lipoprotein particle clearance	2 of 10	2.48	0.0068
GO:0031639	Plasminogen activation	2 of 11	2.44	0.0075
GO:0051649	Establishment of localization in cell	8 of 2375	0.7	0.0078
GO:0033700	Phospholipid efflux	2 of 12	2.4	0.0082
GO:0007596	Blood coagulation	4 of 303	1.3	0.0082
GO:0034380	High-density lipoprotein particle assembly	2 of 13	2.36	0.0084
GO:0044070	Regulation of anion transport	3 of 103	1.64	0.0084
GO:0010769	Regulation of cell morphogenesis involved in differentiation	4 of 309	1.29	0.0084
GO:0042221	Response to chemical	10 of 4333	0.54	0.0084
GO:2001237	Negative regulation of extrinsic apoptotic signaling pathway	3 of 106	1.63	0.0086
GO:0044092	Negative regulation of molecular function	6 of 1163	0.89	0.0098
GO:0030198	Extracellular matrix organization	4 of 338	1.25	0.0104
GO:0032368	Regulation of lipid transport	3 of 116	1.59	0.0105
GO:0034116	Positive regulation of heterotypic cell-cell adhesion	2 of 16	2.27	0.0107
GO:0043691	Reverse cholesterol transport	2 of 17	2.25	0.0118
GO:0043687	Post-translational protein modification	4 of 360	1.22	0.0123
GO:0051241	Negative regulation of multicellular organismal process	6 of 1231	0.87	0.0123
GO:0034375	High-density lipoprotein particle remodeling	2 of 18	2.22	0.0125
GO:0010038	Response to metal ion	4 of 367	1.21	0.0127
GO:0010544	Negative regulation of platelet activation	2 of 19	2.2	0.0135
GO:0050680	Negative regulation of epithelial cell proliferation	3 of 131	1.54	0.0135
GO:0010875	Positive regulation of cholesterol efflux	2 of 20	2.18	0.0144
GO:0042730	Fibrinolysis	2 of 20	2.18	0.0144
GO:0065003	Protein-containing complex assembly	6 of 1293	0.84	0.0146
GO:0097746	Regulation of blood vessel diameter	3 of 139	1.51	0.0151
GO:0043086	Negative regulation of catalytic activity	5 of 807	0.97	0.0169
GO:0031103	Axon regeneration	2 of 23	2.12	0.0171

GO:0031103	Axon regeneration	2 of 23	2.12	0.0171
GO:0033344	Cholesterol efflux	2 of 23	2.12	0.0171
GO:0051592	Response to calcium ion	3 of 150	1.48	0.0173
GO:0002697	Regulation of immune effector process	4 of 418	1.16	0.0174
GO:0051094	Positive regulation of developmental process	6 of 1389	0.81	0.0190
GO:0007584	Response to nutrient	3 of 160	1.45	0.0196
GO:0031667	Response to nutrient levels	4 of 449	1.13	0.0212
GO:0051346	Negative regulation of hydrolase activity	4 of 450	1.13	0.0212
GO:0032369	Negative regulation of lipid transport	2 of 29	2.02	0.0221
GO:2000352	Negative regulation of endothelial cell apoptotic process	2 of 29	2.02	0.0221
GO:1903792	Negative regulation of anion transport	2 of 30	2.0	0.0221
GO:0050776	Regulation of immune response	5 of 896	0.92	0.0221
GO:0010033	Response to organic substance	8 of 3011	0.6	0.0221
GO:0050896	Response to stimulus	12 of 8046	0.35	0.0221
GO:0051050	Positive regulation of transport	5 of 923	0.91	0.0233
GO:0045907	Positive regulation of vasoconstriction	2 of 32	1.97	0.0236
GO:0006898	Receptor-mediated endocytosis	3 of 184	1.39	0.0236
GO:0048584	Positive regulation of response to stimulus	7 of 2257	0.67	0.0252
GO:0060284	Regulation of cell development	5 of 956	0.9	0.0256
GO:0043537	Negative regulation of blood vessel endothelial cell migration	2 of 35	1.93	0.0260
GO:0070328	Triglyceride homeostasis	2 of 35	1.93	0.0260
GO:0019915	Lipid storage	2 of 37	1.91	0.0281
GO:0090207	Regulation of triglyceride metabolic process	2 of 38	1.9	0.0294
GO:1902042	Negative regulation of extrinsic apoptotic signaling pathway...	2 of 39	1.89	0.0306
GO:0070374	Positive regulation of erk1 and erk2 cascade	3 of 209	1.33	0.0306
GO:0031348	Negative regulation of defense response	3 of 212	1.33	0.0314
GO:0043410	Positive regulation of mapk cascade	4 of 543	1.04	0.0317
GO:0070527	Platelet aggregation	2 of 42	1.86	0.0336
GO:0010720	Positive regulation of cell development	4 of 556	1.03	0.0336
GO:1902533	Positive regulation of intracellular signal transduction	5 of 1041	0.86	0.0336
GO:0001937	Negative regulation of endothelial cell proliferation	2 of 43	1.85	0.0339
GO:0032501	Multicellular organismal process	11 of 6933	0.38	0.0339
GO:0051180	Vitamin transport	2 of 46	1.82	0.0376
GO:0006820	Anion transport	4 of 593	1.01	0.0399
GO:0044057	Regulation of system process	4 of 592	1.01	0.0399
GO:0022603	Regulation of anatomical structure morphogenesis	5 of 1095	0.84	0.0399
GO:0034097	Response to cytokine	5 of 1101	0.83	0.0402
GO:0010951	Negative regulation of endopeptidase activity	3 of 248	1.26	0.0432
GO:0051049	Regulation of transport	6 of 1776	0.71	0.0436
GO:1902531	Regulation of intracellular signal transduction	6 of 1807	0.7	0.0460
GO:0032502	Developmental process	10 of 5841	0.41	0.0471
GO:0030449	Regulation of complement activation	2 of 56	1.73	0.0492

(B)

	Molecular Function (Gene Ontology)			
GO-term	description	count in network	strength	false discovery rate
GO:0071813	Lipoprotein particle binding	3 of 29	2.19	0.0036
GO:0008201	Heparin binding	4 of 172	1.54	0.0046
GO:0060228	Phosphatidylcholine-sterol o-acyltransferase activator activity	2 of 6	2.7	0.0092
GO:0005201	Extracellular matrix structural constituent	3 of 119	1.58	0.0298
GO:0120020	Cholesterol transfer activity	2 of 21	2.16	0.0413

Figure S3. STRING functional analysis in analysed network created for differently abundant proteins between two experimental groups Staphylococci and Streptococci enumerated in Table 1. 13 proteins were found (gene names MGC151921, IGL, GLYCAM1, LOC100297192, LOC525947, MGC137014 were not found). Panel A represents biological processes and Panel B molecular function. Gene ontology terms are sorted by false discovery rate, FDR<0.05.

(A)

Cellular Component (Gene Ontology)				
GO-term	description	count in network	strength	false discovery rate
GO:0072562	Blood microparticle	6 of 115	1.9	1.38e-07
GO:0062023	Collagen-containing extracellular matrix	7 of 396	1.42	1.20e-06
GO:0070062	Extracellular exosome	11 of 2099	0.9	1.20e-06
GO:0005788	Endoplasmic reticulum lumen	6 of 308	1.47	6.15e-06
GO:0005577	Fibrinogen complex	3 of 8	2.75	7.23e-06
GO:0034774	Secretory granule lumen	6 of 324	1.45	7.23e-06
GO:0031982	Vesicle	12 of 3879	0.67	7.23e-06
GO:0005576	Extracellular region	12 of 4166	0.64	1.20e-05
GO:0031093	Platelet alpha granule lumen	4 of 68	1.95	1.42e-05
GO:0009897	External side of plasma membrane	5 of 331	1.36	0.00017
GO:0034365	Discoidal high-density lipoprotein particle	2 of 3	3.0	0.00038
GO:0009986	Cell surface	6 of 824	1.04	0.00068
GO:0034363	Intermediate-density lipoprotein particle	2 of 6	2.7	0.00092
GO:0005783	Endoplasmic reticulum	7 of 1438	0.86	0.0010
GO:0031410	Cytoplasmic vesicle	8 of 2386	0.7	0.0024
GO:0034362	Low-density lipoprotein particle	2 of 13	2.36	0.0027
GO:0042627	Chylomicron	2 of 13	2.36	0.0027
GO:0034361	Very-low-density lipoprotein particle	2 of 20	2.18	0.0055
GO:0071682	Endocytic vesicle lumen	2 of 20	2.18	0.0055
GO:0030662	Coated vesicle membrane	3 of 176	1.41	0.0103
GO:0005886	Plasma membrane	10 of 5314	0.45	0.0134
GO:0012505	Endomembrane system	9 of 4542	0.47	0.0241
GO:0012507	ER to Golgi transport vesicle membrane	2 of 58	1.72	0.0299
GO:0031526	Brush border membrane	2 of 58	1.72	0.0299
GO:0030139	Endocytic vesicle	3 of 301	1.18	0.0373

(B)

Subcellular localization (COMPARTMENTS)				
compartment	description	count in network	strength	false discovery rate
GOCC:0072562	Blood microparticle	6 of 116	1.89	1.98e-07
GOCC:0005788	Endoplasmic reticulum lumen	6 of 150	1.78	4.40e-07
GOCC:0005577	Fibrinogen complex	4 of 19	2.5	7.79e-07
GOCC:0005615	Extracellular space	9 of 985	1.14	7.79e-07
GOCC:0005576	Extracellular region	11 of 2035	0.91	7.79e-07
GOCC:1903561	Extracellular vesicle	7 of 444	1.38	1.93e-06
GOCC:0070062	Extracellular exosome	6 of 368	1.39	2.07e-05
GOCC:0031093	Platelet alpha granule lumen	4 of 68	1.95	2.94e-05
GOCC:0030141	Secretory granule	7 of 704	1.18	2.94e-05
GOCC:0034774	Secretory granule lumen	5 of 230	1.51	5.47e-05
GOCC:0009897	External side of plasma membrane	4 of 104	1.76	8.70e-05
GOCC:0031982	Vesicle	9 of 2051	0.82	0.00010
GOCC:0031410	Cytoplasmic vesicle	8 of 1709	0.85	0.00038
GOCC:0070013	Intracellular organelle lumen	9 of 2812	0.68	0.0012
GOCC:0034365	Discoidal high-density lipoprotein particle	2 of 7	2.63	0.0014
GOCC:0034363	Intermediate-density lipoprotein particle	2 of 8	2.58	0.0017
GOCC:0062136	Low-density lipoprotein receptor complex	2 of 8	2.58	0.0017
GOCC:0012505	Endomembrane system	9 of 3080	0.64	0.0019
GOCC:0042627	Chylomicron	2 of 13	2.36	0.0033
GOCC:0005886	Plasma membrane	9 of 3531	0.58	0.0054
GOCC:0034361	Very-low-density lipoprotein particle	2 of 21	2.16	0.0074
GOCC:0034362	Low-density lipoprotein particle	2 of 21	2.16	0.0074
GOCC:0071682	Endocytic vesicle lumen	2 of 22	2.14	0.0074
GOCC:0030662	Coated vesicle membrane	3 of 150	1.48	0.0079
GOCC:0012507	ER to Golgi transport vesicle membrane	2 of 47	1.81	0.0253
GOCC:0005769	Early endosome	3 of 242	1.27	0.0265
GOCC:0030139	Endocytic vesicle	3 of 256	1.25	0.0298

Figure S4. STRING functional analysis in analysed network created for differently abundant proteins between two experimental groups Staphylococci and Streptococci enumerated in

Table S1. 13 proteins were found (gene names MGC151921, IGL, GLYCAM1, LOC100297192, LOC525947, MGC137014 were not found). Panel A shows cellular component and Panel B subcellular localization. Gene ontology terms are sorted by false discovery rate, FDR<0.05.
